# An isothermal DNA amplification method for detection of *Onchocerca volvulus* infection in skin biopsies

**DOI:** 10.1186/s13071-016-1913-7

**Published:** 2016-12-01

**Authors:** Ole Lagatie, Michelle Merino, Linda Batsa Debrah, Alexander Y. Debrah, Lieven J. Stuyver

**Affiliations:** 1 Janssen Diagnostics, Janssen R&D, Turnhoutseweg 30, 2340 Beerse, Belgium; 2 Kumasi Centre for Collaborative Research into Tropical medicine, Kwame Nkrumah University of Science and Technology, Kumasi, Ghana; 3 Faculty of Allied Health Sciences, Kwame Nkrumah University of Science and Technology, Kumasi, Ghana

**Keywords:** *Onchocerca volvulus*, River blindness, Onchocerciasis, Skin biopsy, DNA, *cox*1, LAMP, Isothermal, Diagnostic

## Abstract

**Background:**

Diagnostic procedures for the diagnosis of infection with the nematode parasite *Onchocerca volvulus* are currently based on the microscopic detection of microfilariae in skin biopsies. Alternative approaches based on amplification of parasitic DNA in these skin biopsies are currently being explored. Mostly this is based on the detection of the O-150 repeat sequence using PCR based techniques.

**Methods:**

An isothermal, loop-mediated amplification method has been designed using the mitochondrial *O. volvulus cox*1 gene as a target.

**Results:**

Analysis of dilution series of synthetic DNA containing the targeted sequence show a non-linear dose-response curve, as is usually the case for isothermal amplification methods. Evaluation of cross-reactivity with the heterologous sequence from the closely related parasites *Wuchereria bancrofti*, *Loa loa* and *Brugia malayi* demonstrated strong specificity, as none of these sequences was amplified. The assay however amplified both *O. volvulus* and *O. ochengi* DNA, but with a different melting point that can be used to discriminate between the species. Evaluation of this assay in a set of skin snip biopsies collected in an endemic area in Ghana showed a high correlation with O-150 qPCR and also demonstrated a similar sensitivity. Compared to qPCR, LAMP had a sensitivity of 88.2% and a specificity of 99.2%.

**Conclusions:**

We have developed a sensitive and specific loop-mediated amplification method for detection of *O. volvulus* DNA in skin biopsies that is capable of providing results within 30 min.

**Electronic supplementary material:**

The online version of this article (doi:10.1186/s13071-016-1913-7) contains supplementary material, which is available to authorized users.

## Background

Onchocerciasis, infection with the filarial nematode *Onchocerca volvulus* is a neglected tropical disease which is best known as river blindness [1, 2]. In Africa at least 120 million people are at risk of infection. The last comprehensive survey conducted in 2008 indicated that 26 million people were infected with *O. volvulus*, of which 265,000 individuals were blind and 746,000 were visually impaired. In addition, approximately four million people suffer from onchodermatitis with severe itching [3]. Presently, treatment is based on microfilaricidal agents, such as ivermectin (Mectizan, Merck), as no approved macrofilaricide drugs or vaccines are available. Since microfilaricides only affect the larval stage of *O. volvulus* with little or no impact on the adult worm, annual or bi-annual treatments for several years are required [4, 5]. Since the start of these mass drug administration programs (MDA) in 1987, ivermectin has been used to treat hundreds of millions of people with a resultant reduction in both visual impairment and symptomatic onchodermatitis [6].

Evaluation of MDA programs, and ultimately also guidance to stop them, is based mainly on monitoring of infection levels in human populations, as well as in its vector, the blackfly of the genus *Simulium*. Besides clinical examination by palpation of nodules formed by adult worms (macrofilariae), diagnostic tools for detection of *O. volvulus* infection involves finding microfilariae (mf) in small, superficial skin biopsy samples (skin snips) using microscopy [7]. The latter can be a challenge, especially when larval densities are low, which is often the case during or the first months after treatment with ivermectin. The sensitivity of this test has been further increased by using polymerase chain reaction based detection of the *Onchocerca* specific O-150 repeat sequence [8–11].

Several efforts have been undertaken to identify novel biomarkers that offer a less-invasive, specific and sensitive marker for infection with *O. volvulus* [12]. The most advanced of these tests, is the rapid-format test for the detection of IgG4 antibodies to the parasitic antigen Ov-16, which is predominantly useful in a surveillance setting [13–18]. Another approach that has shown promise is the use of metabolome analysis of serum or urine samples from infected individuals, which has led to the identification of urinary *N*-acetyltyramine-*O*,β-glucuronide (NATOG) as a unique biomarker for *O. volvulus* infection [19–21]. More recent work has focused on the detection of parasitic microRNAs in the blood of infected individuals, but the low levels of these biomarkers may pose a real challenge to be useful as a diagnostic marker [22–24].

Isothermal amplification provides a simple process that rapidly and efficiently accumulates nucleic acids at constant temperature [25]. In contrast to PCR-based amplification, no temperature cycles are required, which facilitates its integration into microsystems or portable devices. One of the most frequently used isothermal amplification technologies is the loop mediated isothermal amplification (LAMP), which is based on two primer sets that recognized six different sites on the target DNA and an optional third set of primers, so-called loop primers to accelerate the reaction [26, 27]. LAMP offers advantages over other molecular diagnostic methods because it is simple, rapid and highly specific. This technology has been evaluated for the diagnosis of the filarial parasites *Brugia malayi* [28], *Wuchereria bancrofti* [29] and *Loa loa* [30–32]. Also for *Onchocerca volvulus*, a LAMP assay has been developed for surveillance of parasite transmission in the blackfly [33].

In the present study, we report the design of a LAMP assay that targets the mitochondrial encoded *O. volvulus cox*1 gene. This work provides evidence of the high sensitivity of this assay, and the close concordance with qPCR based detection of *O. volvulus* DNA.

## Methods

### Study samples

Skin biopsy samples were collected as part of a field study in Ghana. This study was undertaken in an Onchocerciasis-endemic community located in Adansi South District along the Pra River basins in the Ashanti Region of Ghana. Physical examinations were performed to identify those subjects having palpable nodules. Most subjects were participating in MDA programs with ivermectin. An overview of the patient demographics is provided in Table 1. From each participant two skin biopsies were taken, one from the right and one from the left hip. Immediately after sampling, biopsies were transferred individually into a single round bottom well of a 96-well plate, submerged in saline solution and after overnight incubation at room temperature each biopsy was examined microscopically and the emerged microfilariae of *O. volvulus* counted. [34]. Thereafter the two biopsies collected from each participant were weighed and transferred individually to microcentrifuge tubes and stored in liquid nitrogen.

**Table 1:** Characteristics of study populations used in this study

Characteristic	Group	
	Nodule-positive	Endemic controls
No. of subjects	99	51
Age, median (min-max)	47 (21–85)	35 (18–81)
Gender, *n* (%)		
Male	53 (54)	26 (51)
Female	46 (46)	25 (49)
No. of nodules, median (min-max)	1 (1–5)	0
mf status, *n* (%)		
0 mf/mg	89 (90)	51 (100)
0–5 mf/mg	9 (9)	0 (0)
5–10 mf/mg	1 (1)	0 (0)
No. of IVM rounds, median (min-max)	2 (0–10)	0 (0–1)
Time since last treatment		
Not treated	16 (16)	34 (67)
< 20 months	68 (69)	5 (10)
> 20 months	15 (15)	12 (24)
Ov16 status, *n* (%)		
Positive	68 (69)	26 (51)
Negative	31 (31)	25 (49)

*Abbreviations*: *mf* microfilaria, *Ov16 O. volvulus* antigen with molecular weight of 16 kDa

### Extraction of genomic DNA from skin biopsies

Genomic DNA was extracted from each skin biopsy using the OMEGA E.Z.N.A. tissue DNA kit (Norcross, GA, USA), according to the manufacturer’s instructions. DNA was eluted in 200 μl elution buffer. The quantity and quality of extracted DNA was assessed with a NanoDrop Spectrophotometer (ND-1000, Thermo Scientific, Waltham, MA, USA) and DNA was stored at -20 °C before analysis [34]. Extracted DNA concentrations ranged from 0.2 ng/μl to 134 ng/μl. Extracts were diluted in nuclease free water to a concentration of 10 ng/μl, except for those samples which already had a lower concentration, which were used undiluted. In order to confirm that DNA in the extracts was intact and could be amplified, qPCR targeting the human beta-actin gene was performed on 10 ng of template DNA. This was done using the PrimeTime *actb* qPCR assay, according to the manufacturer’s instructions (Integrated DNA Technologies, Leuven, Belgium). The primers and probe included in the assay were Fwd Primer 5'-CAC GGC TAG CTG TG-3', Rev Primer 5'-ATC GTT CGT TGA GCG ATT AGC AG-3', Probe 5'-6-FAM-GTG GCT CCA TCT TAG CCC TAG T-IBFQ-3' (Iowa Black® FQ), with internal quencher ZEN. Four of the 150 extracts (including 1 mf positive sample) were excluded for further analysis as they did not meet the acceptance criteria described in the statistical analysis below (see Additional file 1: Figure S1).

### Quantitative real-time PCR assay for detecting O-150 DNA

A TaqMan qPCR assay based on a previously published method (see [35]) was used to quantify *O. volvulus* O-150 DNA. The primers and probe included OvFWD 5'-TGT GGA AAT TCA CCT AAA TAT G-3', OvREV 5'-AAT AAC TGA TGA CCT ATG ACC-3', OvProbe 5'-6-FAM-TAG GAC CCA ATT CGA ATG TAT GTA CCC-IBFQ-3' (Iowa Black® FQ), with internal quencher ZEN (Integrated DNA Technologies, Leuven, Belgium). Taqman Universal Master Mix without UNG (Applied Biosystems, P/N 4440040) and nuclease free water were used with all reactions with the following concentrations and volumes: 1 μl of 10 μM OvFWD, 1 μl of 10 μM OvREV, 2.5 μl of 10 μM OvProbe, 10 μl of 2× Master Mix, 5 μl of template DNA from extracted skin biopsies, or 2 μl of linearized Ov-150 positive control plasmid at a concentration of 10^0^ to 10^6^ copies/μl (Integrated DNA Technologies, Leuven, Belgium), and nuclease-free water was added up to a final volume of 20 μl. Reactions were performed in duplicate on a Roche Lightcycler 480 instrument with the following conditions: 95 °C 10 min (95 °C 15 s, 49 °C 15 s, 60 °C 30 s) × 45 cycles. The second derivate method was used to calculate Cq values and samples were considered positive for Ov-150 DNA if Cq (quantification cycle) values were greater than 36.15 (corresponding to 2 log copies/reaction) in both duplicates. The calibration curve (0.3 to 6.3 log copies/reaction) of the O-150 qPCR assay is given by the formula y = -3.57x + 43.29 (Additional file 2: Figure S2).

### Loop-mediated isothermal amplification primer design

The complete mitochondrial genome sequence of *O. volvulus* (NC_001861.1) was retrieved from the NCBI database. LAMP primers targeting *O. volvulus* mitochondrial DNA were designed using LAMP Designer V1.13 (OptiGene, Horsham, UK). Two sets of primers comprising two outer (F3 and B3), and two inner (FIP and BIP) were selected. FIP contained F1c (complementary to F1), and the F2 sequence. BIP contained the B1c sequence (complementary to B1) and the B2 sequence. Additional loop primers, forward loop primer (LoopF) and backward loop primer (LoopB) were included in the reaction. BLAST analysis [36] of the fragment located between the two outer primers indicated the target gene was *cox*1.

### Loop-mediated isothermal amplification assay

LAMP reactions were performed using the primers designed as described above. LAMP reactions were performed in a final volume of 25 μl. Reaction mixtures contained 12 μl Isothermal Mastermix (OptiGene, Horsham, UK), 0.5 μl of each F3 and B3 primer (10 μM), 2 μl of each FIP and BIP primer (20 μM), 1 μl of each LoopF and LoopB (10 μM), 5 μl of template DNA from extracted skin biopsies, or 5 μl of specific gBlocks (*O. volvulus cox*1, *O. ochengi cox*1, *W. bancrofti cox*1, *L. loa cox*1 or *B. malayi cox*1) at a concentration of 10^0^ to 10^6^ copies/μl (Integrated DNA Technologies, Leuven, Belgium), and nuclease-free water was added up to a final volume of 25 μl. The mixture was incubated at 65 °C for 45 min, with fluorescent detection every 30 s on a Roche Lightcycler 480 instrument. At the end of the incubation period, *T*_m_ determination was performed by increasing the temperature to 95 °C with 5 fluorescence acquisitions per °C. The threshold method, using an arbitrarily selected threshold of 10 RFU, was used to calculate Ct values. Time to LAMP was calculated by dividing the obtained Ct values by 2 (one cycle corresponds to 30 s). The detection limit of LAMP was determined to be 2 log copies/reaction as at this concentration a positive signal was reproducibly obtained in the assay. Based on the calibration curves obtained with the synthetic DNA and the higher variation observed in Time to LAMP at low concentrations (2 log copies/reaction), a cut-off of 30 min was defined. Samples were considered positive if average Time to LAMP < 30 min.

### Statistical analysis

Results of human Actin qPCR were subjected to statistical analysis in order to identify samples with poor extraction and/or qPCR efficiency. Average Cq value and standard deviation of all samples was calculated and possible outliers were identified as samples with Cq value > average + 2 standard deviations, SD. In case outliers were detected, samples were not included for further analysis. For comparison of *T*_m_ values in different groups, a two-tailed unpaired *t*-test with 95% confidence interval was performed. *P*-values < 0.05 were considered to be significantly different. Correlation between different parameters was analyzed using linear regression. *P*-values were calculated to determine whether slope was significantly non-zero and the strength of correlation was determined using *R* ^2^-value. All statistical analyses were performed using GraphPad Prism version 6.02.

## Results

**Fig. 1:**
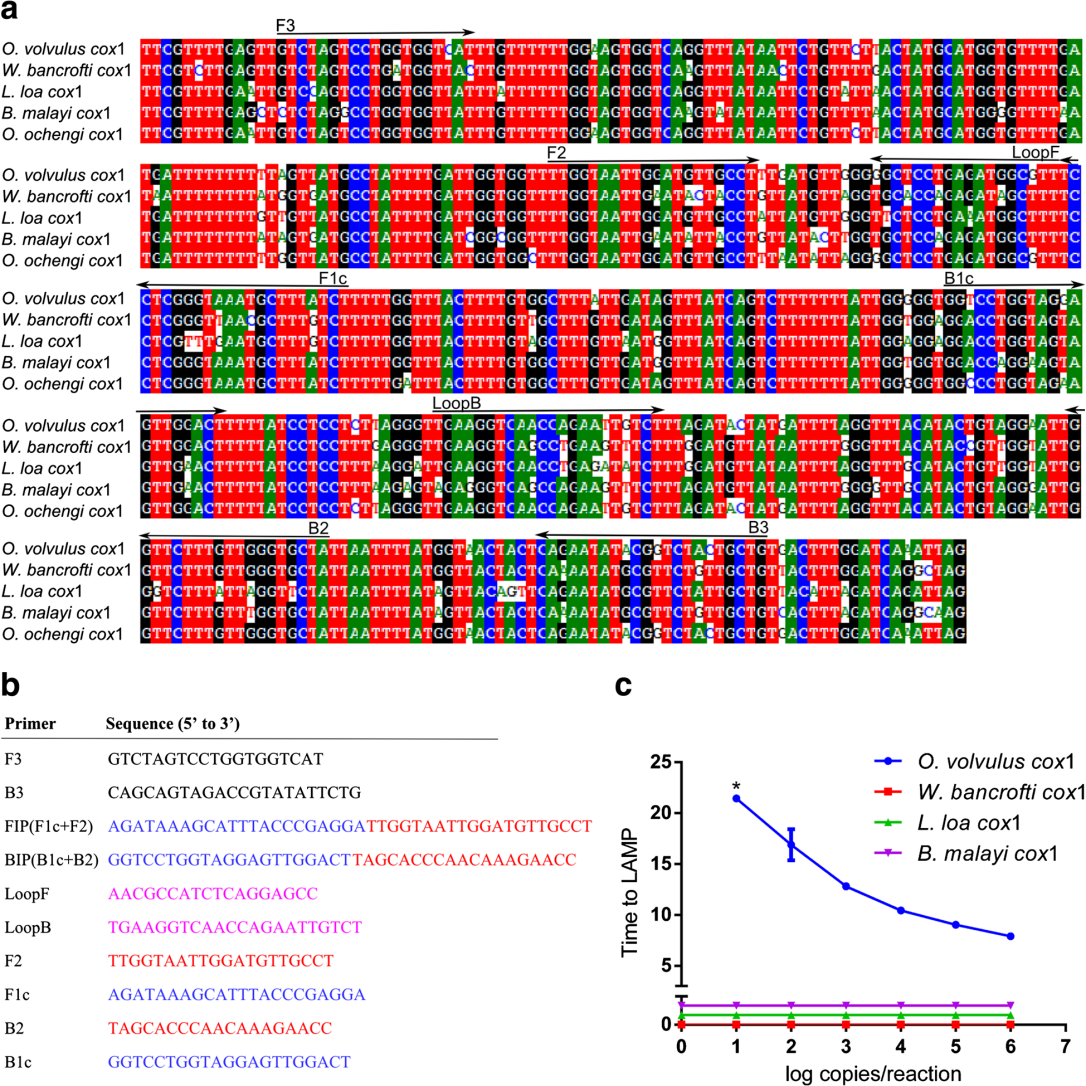
Design of a LAMP assay targeting *O. volvulus cox*1. **a** Alignment of partial gene sequences of cytochrome *c* oxidase subunit 1 from *O. volvulus*, *W. bancrofti*, *L. loa*, *B. malayi* and *O. ochengi*. **b** Primer set targeting *O. volvulus cox*1. **c** Species-specific LAMP assay targeting *O. volvulus cox*1. **c** Dilution series of gBlocks containing the *cox*1 fragment of *O. volvulus*, *W. bancrofti*, *L. loa* and *B. malayi* were used as template in the LAMP assay using real-time fluorescent dye detection. All samples were analyzed in duplicate. Dilution indicated with an asterisk had detectable signal in only one of both duplicates

A primer set targeting *O. volvulus cox*1 was designed using LAMP Designer software (Fig. 1a, b). LAMP assays were performed on a dilution series of synthetic DNA containing the target sequence. Since there is a close sequence homology with other helminth parasites (Fig. 1a), assays were also performed on dilution series of synthetic DNA containing the heterologous sequence from *Wuchereria bancrofti*, *Loa loa* and *Brugia malayi* in order to determine the specificity of the primer set (Fig. 1c). The region heterologous to the *O. volvulus* region targeted by the assay (i.e. from F3 to B3 primer) was 87.0, 86.7 and 86.2% identical to sequences from *W. bancrofti*, *L. loa* and *B. malayi*, respectively. When the *O. volvulus* target sequence was used as a template, a clear amplification was observed, with concentration-dependent time to reach threshold fluorescence signal, whereas no amplification was observed within the time interval examined (45 min) when the same amount of heterologous DNA from *W. bancrofti*, *L. loa* or *B. malayi* was used.

**Fig. 2:**
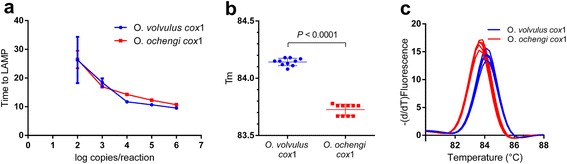
*O. volvulus cox*1 assay discriminates between *O. volvulus* and *O. ochengi*. **a** Dilution series of gBlocks containing the *cox*1 fragment of *O. volvulus* and *O. ochengi* were used as template in the *O. volvulus cox*1 LAMP assay using real-time fluorescent dye detection. **b** Melting point determination of the amplicons generated from different dilutions of *O. volvulus* and *O. ochengi* template. **c** Melting curves of the amplicons generated from *O. volvulus* and *O. ochengi* template

Specificity of the assay was further investigated by examining the potential of the primer set to amplify the heterologous region of *O. ochengi*. Similarity between the *O. volvulus cox*1 and *O. ochengi cox*1 is very high, with only 10 nucleotide differences (i.e. 97.5% identity) over the entire sequence targeted by the assay (Fig. 1a). Consequently, both synthetic DNA fragments containing the respective target sequences are amplified with a similar efficiency and a same time to LAMP as a result (Fig. 2a). The few nucleotides difference does however result in a statistically different melting point (*T*_m_). Mean melt temperatures of 84.14 ± 0.03 and 83.73 ± 0.03 °C for *O. volvulus cox*1 and *O. ochengi cox*1, respectively (*t*_(18)_ = 22.56, *P* < 0.0001, Fig. 2b), were determined across 10 reactions. Melting curves obtained on both DNA fragments were largely overlapping (Fig. 2c).

**Fig. 3:**
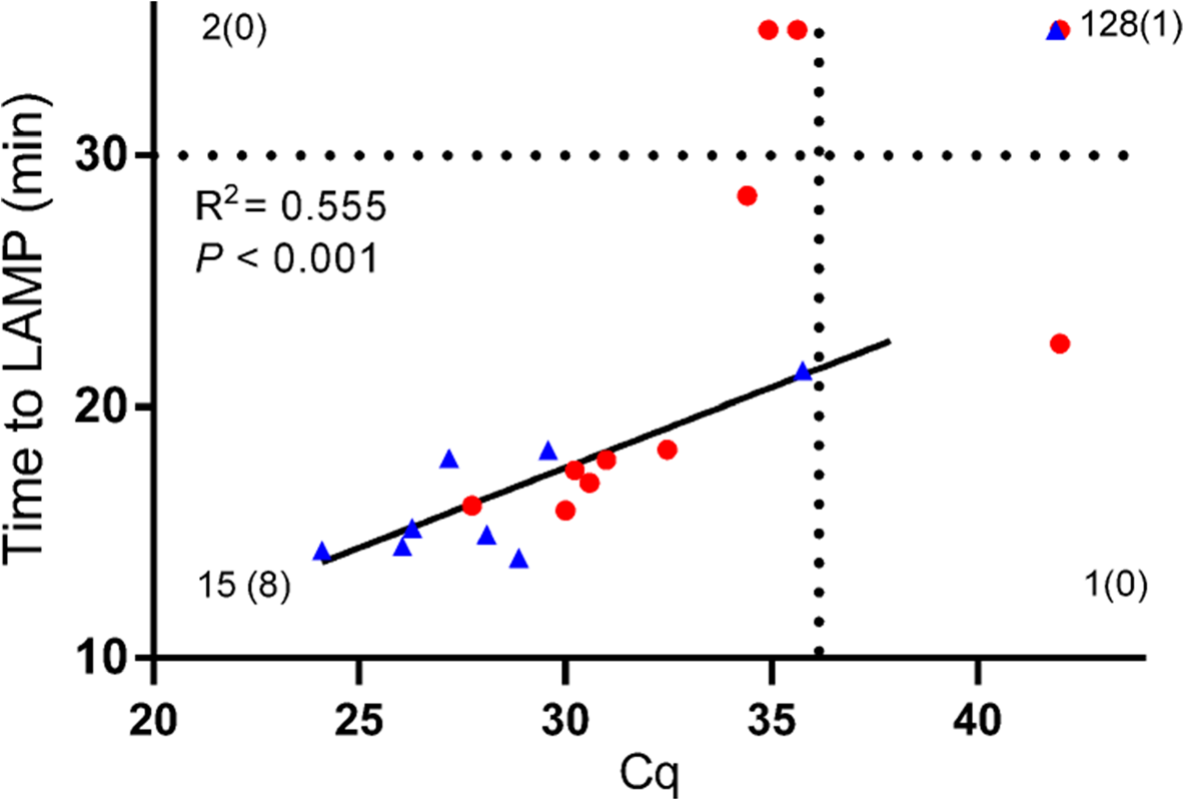
Comparison of results obtained by LAMP, O-150 qPCR and microscopic detection of microfilariae in skin snips. Comparison of results obtained by *O. volvulus cox*1 LAMP and O-150 qPCR for 146 subjects. Samples that were positive in microscopic examination are indicated in blue (*n* = 9). For each quadrant the total number of samples is indicated, as well as the number of microscopy positive samples in parentheses

To demonstrate the applicability of the *O. volvulus cox*1 LAMP assay to determine active *O. volvulus* infection in individuals living in endemic areas, skin biopsy samples were collected in the Ashanti Region, an onchocerciasis endemic region in Ghana. A total of 150 individuals, of which 99 had obvious nodules and 51 had no signs of onchocerciasis, were included in this study. Of the 99 nodule-positive individuals, only 10 were found to contain microfilariae upon microscopic examination. Total genomic DNA was isolated from these samples. From the 150 gDNA samples, 4 were excluded as they did not meet the acceptance criteria (see Methods). The 146 analysed extracts were tested for the presence of *O. volvulus* DNA using both qPCR and LAMP (Fig. 3a and Table 2). While only 9 individuals were found to be test-positive by microscopy, 17 were positive by qPCR and 15 by LAMP. This observation confirms the superior sensitivity of molecular detection over microscopic examination. These data also indicate a sensitivity of LAMP of 88.2% compared to qPCR (Table 3). The 2 samples negative in LAMP but positive in qPCR had Cq values of 35 and above, indicating they contained extremely low amounts of *O. volvulus* DNA. One sample that was found to be negative in qPCR was positive in LAMP, corresponding to a specificity of 99.2% as compared to qPCR. Also, a clear correlation could be observed between Cq values obtained during qPCR and the Time to LAMP obtained in the LAMP assay (*R* ^2^ = 0.555, *P* < 0.001).

**Table 2:** Cross tabulation of results obtained by qPCR, LAMP, microscopic detection of micro-filariae (mf) in skin snips, and nodule palpation for 146 individuals in the Adansi South District, Ghana

	Nodule-positive		Nodule-negative		
	mf-positive	mf-negative	mf-positive	mf-negative	Totals
qPCR-positive	8	5	0	4	17
qPCR-negative	1	81	0	47	129
LAMP-positive	8	4	0	4	16
LAMP-negative	1	82	0	47	130
Totals	9	86	0	51	146

*Abbreviation*: *mf* microfilariae

**Table 3:** Determination of sensitivity and specificity of LAMP as compared to qPCR

	qPCR-positive	qPCR-negative	
LAMP-positive	15	1	PPV 93.8%
LAMP-negative	2	128	NPV 98.5%
	Sensitivity: 88.2%	Specificity: 99.2%	

## Discussion

This work reports the development of a LAMP method for detection of *O. volvulus* DNA and its use in field collected skin biopsies from an onchocerciasis endemic region in Ghana. Currently, diagnosis of infection with *O. volvulus* is predominantly based on nodule palpation and microscopic detection of microfilariae in superficial skin biopsies [12]. The latter test is particularly useful for detection of active infection, but consequently suffers from insufficient sensitivity in an MDA era where the load of living microfilariae in the skin is dramatically reduced [37, 38]. Previous studies already demonstrated that the detection of *O. volvulus* DNA using PCR in such skin biopsies has a higher sensitivity than the classical microscopy approach [8]. Sensitivity was further improved by development of a probe-based qPCR assay [11]. qPCR also has the advantage that it is relatively fast and is less prone to contamination.

Results presented here demonstrate that similar sensitivities can be achieved with the *O. volvulus cox*1 LAMP assay. Only those samples containing very low levels of *O. volvulus* DNA (Cq values in qPCR > 35) were found to have a negative result in LAMP. Importantly, samples that were found to be positive upon microscopic examination were confirmed to be positive using qPCR and LAMP in eight out of nine cases. Although the O-150 qPCR assay has the advantage of targeting a repeat sequence that is present multiple times in the *O. volvulus* genome, the *cox*1 LAMP assay has a comparable clinical sensitivity of 88.2%. A likely explanation for this high sensitivity is the fact that the *cox*1 gene is located in the mitochondrial genome, while O-150 is located in the nuclear genome [39, 40]. Since every cell contains hundreds of mitochondrial DNA copies, it makes mitochondrial encoded sequences ideal targets for molecular detection requiring high sensitivity, as is the case for *O. volvulus* detection in skin biopsies [41]. For this reason, the *cox*1 gene has already been used as a target for other molecular assays to detect the presence of the jellyfish *Cyanea nozakii* in seawater samples or the presence of the olive tree pest *Bactrocera oleae* in the guts of arthropods [42, 43]. It will be of interest for future work to study whether a correlation exists between number of mf as determined by microscopy and time to LAMP. Sample collections from infected, but untreated patients will be essential for this as these samples should display a broader range of microfilaremia required to properly assess such correlation.

The LAMP assay presented here is also shown to be highly specific for *Onchocerca* species as no amplification could be observed when the heterologous DNA from *W. bancrofti*, *L. loa* and *B. malayi* were used as template in the reaction. However, the assay also detects *O. ochengi*, but with a slight difference in *T*_m_ of the amplicons produced. As there is a large overlap in the melting curves obtained for both species, *T*_m_ determination will be able to distinguish both species in case samples contain only DNA from one of both species but not in cases were both are present in one and the same sample. This cross-reactivity with *O. ochengi* DNA is not expected to pose an issue when used to analyze skin biopsies from humans as this species is typically present in cattle and not in humans [44]. As a consequence of this cross-reactivity this assay might not be ideally suited for specific detection of *O. volvulus* in *Simulium* blackfly vectors where it may be used to assess changes in parasite prevalence during or after MDA programs [45–49].

The main advantages of isothermal amplification methods, such as LAMP, over PCR-based detection techniques, are the speed with which the assays can be completed, and the simple and relatively inexpensive equipment required [26, 50]. The *O. volvulus cox*1 LAMP assay we developed here reaches threshold fluorescence after just 10 min in cases of high target concentration and can take up to 30 min for low concentration samples. This characteristic, together with the availability of inexpensive reagents and the possibility to use a portable real time machine for monitoring the LAMP amplification, is of great importance for its use for point of care nucleic acid based diagnosis [51].

One major challenge of the analysis of *O. volvulus* DNA skin biopsies remains its invasive nature and the requirement of extraction techniques to isolate the parasitic DNA from this tissue sample [12]. Several efforts have been undertaken to evaluate simple extraction methods that are compatible with LAMP, of which boiling in 5% Chelex buffer or in NaOH appear to be the most promising approaches [52, 53]. Interestingly, extraction and LAMP detection have been integrated in single devices for detection of e.g. *Mycobacterium tuberculosis* in sputum samples [54]. Whether isolation of *O. volvulus* DNA from skin biopsies also can be achieved with high efficiency using similar extraction methods, will need to be further investigated.

## Conclusions

We have developed a sensitive and specific loop-mediated amplification method for detection of *O. volvulus* DNA in skin biopsies that is capable of providing results within 30 min.

## Additional files

Additional file 1: Figure S1. Cq values of *actb* qPCR assay on the 150 gDNA samples. Mean and standard deviation are indicated, as well as the line indicating mean + 2 standard deviations. The four samples that had Cq values above this cut-off were excluded in further analyses. (PDF 29 kb)
Additional file 2: Figure S2. Calibration curve of the O-150 qPCR assay. Calibration samples have been analyzed in 4-fold. Geometric mean and 95% confidence interval are indicated. (PDF 34 kb)

## Abbreviations

*actb*: Beta-actin
Cq: Quantification cycle
Ct: Threshold cycle
LAMP: Loop-mediated isothermal amplification
MDA: Mass drug administration
mf: Microfilariae
NATOG: *N*-acetyltyramine-*O*,β-glucuronide
qPCR: Quantitative real-time PCR
*T*_m_: Melting temperature
UNG: Uracil N-glycosylase

## References

[1] Enk CD (2006). Onchocerciasis - river blindness. Clin Dermatol.

[2] Borup LH, Peters JS, Sartori CR (2003). Onchocerciasis (river blindness). Cutis.

[3] World Health Organisation (2010). Working to overcome the global impact of neglected tropical diseases.

[4] Diawara L, Traore MO, Badji A, Bissan Y, Doumbia K, Goita SF (2009). Feasibility of onchocerciasis elimination with ivermectin treatment in endemic foci in Africa: first evidence from studies in Mali and Senegal. PLoS Negl Trop Dis.

[5] Traore MO, Sarr MD, Badji A, Bissan Y, Diawara L, Doumbia K (2012). Proof-of-principle of onchocerciasis elimination with ivermectin treatment in endemic foci in Africa: final results of a study in Mali and Senegal. PLoS Negl Trop Dis.

[6] World Health Organisation. African Programme for Onchocerciasis Control: progress report, 2014-2015. Weekly epidemiological record/Health Section of the Secretariat of the League of Nations. 2015;90(49):661-674.

[7] Taylor HR, Munoz B, Keyvan-Larijani E, Greene BM (1989). Reliability of detection of microfilariae in skin snips in the diagnosis of onchocerciasis. Am J Trop Med.

[8] Toe L, Boatin BA, Adjami A, Back C, Merriweather A, Unnasch TR (1998). Detection of *Onchocerca volvulus* infection by O-150 polymerase chain reaction analysis of skin scratches. J Infect Dis.

[9] Fink DL, Fahle GA, Fischer S, Fedorko DF, Nutman TB (2011). Toward molecular parasitologic diagnosis: enhanced diagnostic sensitivity for filarial infections in mobile populations. J Clin Microbiol.

[10] Boatin BA, Toe L, Alley ES, Nagelkerke NJ, Borsboom G, Habbema JD (2002). Detection of *Onchocerca volvulus* infection in low prevalence areas: a comparison of three diagnostic methods. Parasitology.

[11] Lloyd MM, Gilbert R, Taha NT, Weil GJ, Meite A, Kouakou IM, Fischer PU (2015). Conventional parasitology and DNA-based diagnostic methods for onchocerciasis elimination programmes. Acta Trop.

[12] Vlaminck J, Fischer PU, Weil GJ (2015). Diagnostic tools for onchocerciasis elimination programs. Trends Parasitol.

[13] Lipner EM, Dembele N, Souleymane S, Alley WS, Prevots DR, Toe L (2006). Field applicability of a rapid-format anti-Ov-16 antibody test for the assessment of onchocerciasis control measures in regions of endemicity. J Infect Dis.

[14] Weil GJ, Steel C, Liftis F, Li BW, Mearns G, Lobos E, Nutman TB (2000). A rapid-format antibody card test for diagnosis of onchocerciasis. J Infect Dis.

[15] Steel C, Golden A, Stevens E, Yokobe L, Domingo GJ, de Los Santos T, Nutman TB. Rapid point-of-contact tool for mapping and integrated surveillance of *Wuchereria bancrofti* and *Onchocerca volvulus* infection. Clin Vaccine Immunol. 2015;22(8):896–901. 10.1128/CVI.00227-15 PMC4519720 26018537

[16] Golden A, Steel C, Yokobe L, Jackson E, Barney R, Kubofcik J (2013). Extended result reading window in lateral flow tests detecting exposure to *Onchocerca volvulus*: a new technology to improve epidemiological surveillance tools. PLoS One.

[17] Lavebratt C, Dalhammar G, Adamafio NA, Nykanen-Dejerud U, Mingarini K, Ingemarsson K (1994). A simple dot blot assay adaptable for field use in the diagnosis of onchocerciasis: preparation of an adult worm antigen fraction which enhances sensitivity and specificity. Trans R Soc Trop Med Hyg.

[18] Chandrashekar R, Ogunrinade AF, Weil GJ (1996). Use of recombinant *Onchocerca volvulus* antigens for diagnosis and surveillance of human onchocerciasis. Trop Med Int Health.

[19] Globisch D, Moreno AY, Hixon MS, Nunes AAK, Denery JR, Specht S (2013). *Onchocerca volvulus*-neurotransmitter tyramine is a biomarker for river blindness. Proc Natl Acad Sci U S A.

[20] Denery JR, Nunes AA, Hixon MS, Dickerson TJ, Janda KD. Metabolomics-based discovery of diagnostic biomarkers for onchocerciasis. PLoS Negl Trop Dis. 2010;4(10):e834. 10.1371/journal.pntd.0000834 PMC2950146 20957145

[21] Lagatie O, Njumbe Ediage E, Batsa Debrah L, Diels L, Nolten C, Vinken P, et al. Evaluation of the diagnostic potential of urinary N-Acetyltyramine-O, beta-glucuronide (NATOG) as diagnostic biomarker for *Onchocerca volvulus* infection. Parasit Vectors. 2016;9(1):302. 10.1186/s13071-016-1582-6 PMC4877973 27216752

[22] Tritten L, O’Neill M, Nutting C, Wanji S, Njouendoui A, Fombad F (2014). *Loa loa* and *Onchocerca ochengi* miRNAs detected in host circulation. Mol Biochem Parasitol.

[23] Tritten L, Burkman E, Moorhead A, Satti M, Geary J, Mackenzie C, Geary T (2014). Detection of circulating parasite-derived microRNAs in filarial infections. PLoS Negl Trop Dis.

[24] Quintana JF, Makepeace BL, Babayan SA, Ivens A, Pfarr KM, Blaxter M (2015). Extracellular *Onchocerca*-derived small RNAs in host nodules and blood. Parasit Vectors.

[25] Zhao Y, Chen F, Li Q, Wang L, Fan C (2015). Isothermal amplification of nucleic acids. Chem Rev.

[26] Notomi T, Mori Y, Tomita N, Kanda H (2015). Loop-mediated isothermal amplification (LAMP): principle, features, and future prospects. J Microbiol.

[27] Notomi T, Okayama H, Masubuchi H, Yonekawa T, Watanabe K, Amino N, Hase T (2000). Loop-mediated isothermal amplification of DNA. Nucleic Acids Res.

[28] Poole CB, Tanner NA, Zhang Y, Evans TC, Carlow CK (2012). Diagnosis of brugian filariasis by loop-mediated isothermal amplification. PLoS Negl Trop Dis.

[29] Takagi H, Itoh M, Kasai S, Yahathugoda TC, Weerasooriya MV, Kimura E (2011). Development of loop-mediated isothermal amplification method for detecting *Wuchereria bancrofti* DNA in human blood and vector mosquitoes. Parasitol Int.

[30] Poole CB, Ettwiller L, Tanner NA, Evans Jr TC, Wanji S, Carlow CK. Genome filtering for New DNA biomarkers of *Loa loa* infection suitable for loop-mediated isothermal amplification. PLoS One. 2015;10(9):e0139286. 10.1371/journal.pone.0139286 PMC4586141 26414073

[31] Fernandez-Soto P, Mvoulouga PO, Akue JP, Aban JL, Santiago BV, Sanchez MC, Muro A (2014). Development of a highly sensitive loop-mediated isothermal amplification (LAMP) method for the detection of *Loa loa*. PLoS One.

[32] Drame PM, Fink DL, Kamgno J, Herrick JA, Nutman TB (2014). Loop-mediated isothermal amplification for rapid and semiquantitative detection of *Loa loa* infection. J Clin Microbiol.

[33] Alhassan A, Makepeace BL, LaCourse EJ, Osei-Atweneboana MY, Carlow CK (2014). A simple isothermal DNA amplification method to screen black flies for *Onchocerca volvulus* infection. PLoS One.

[34] Debrah AY, Specht S, Klarmann-Schulz U, Batsa L, Mand S, Marfo-Debrekyei Y, et al. Doxycycline leads to sterility and enhanced killing of female *Onchocerca volvulus* worms in an area with persistent microfilaridermia after repeated ivermectin treatment: a randomized, placebo-controlled, Double-Blind Trial. Clin Infect Dis. 2015;61(4):517–26. 10.1093/cid/civ363 PMC4518165 25948064

[35] Golden A, Faulx D, Kalnoky M, Stevens E, Yokobe L, Peck R (2016). Analysis of age-dependent trends in Ov16 IgG4 seroprevalence to onchocerciasis. Parasit Vectors.

[36] Altschul SF, Gish W, Miller W, Myers EW, Lipman DJ (1990). Basic local alignment search tool. J Mol Biol.

[37] Boussinesq M, Prod’hon J, Chippaux JP, Quillevere D (1993). Long-term effect of a single dose of ivermectin on skin microfilarial density in an endemic onchocerciasis area of North Cameroon. Ann Soc Belg Med Trop.

[38] Bottomley C, Isham V, Vivas-Martinez S, Kuesel AC, Attah SK, Opoku NO, et al. Modelling neglected tropical diseases diagnostics: the sensitivity of skin snips for *Onchocerca volvulus* in near elimination and surveillance settings. Parasit Vectors. 2016;9(1):343. 10.1186/s13071-016-1605-3 PMC4908809 27301567

[39] Ferri E, Barbuto M, Bain O, Galimberti A, Uni S, Guerrero R (2009). Integrated taxonomy: traditional approach and DNA barcoding for the identification of filarioid worms and related parasites (Nematoda). Front Zool.

[40] Lefoulon E, Bain O, Bourret J, Junker K, Guerrero R, Canizales I (2015). Shaking the tree: multi-locus sequence typing usurps current onchocercid (filarial nematode) phylogeny. PLoS Negl Trop Dis.

[41] Robin ED, Wong R (1988). Mitochondrial DNA molecules and virtual number of mitochondria per cell in mammalian cells. J Cell Physiol.

[42] Liu Z, Dong Z, Liu D (2016). Development of a rapid assay to detect the jellyfish *Cyanea nozakii* using a loop-mediated isothermal amplification method. Mitochondrial DNA A DNA Mapp Seq Anal.

[43] Rejili M, Fernandes T, Dinis AM, Pereira JA, Baptista P, Santos SA, Lino-Neto T (2016). A PCR-based diagnostic assay for detecting DNA of the olive fruit fly, *Bactrocera oleae*, in the gut of soil-living arthropods. Bull Entomol Res.

[44] Bwangamoi O (1969). *Onchocerca ochengi* new species, an intradermal parasite of cattle in East Africa. Bull Epizoot Dis Afr.

[45] Katholi CR, Toe L, Merriweather A, Unnasch TR (1995). Determining the prevalence of *Onchocerca volvulus* infection in vector populations by polymerase chain reaction screening of pools of black flies. J Infect Dis.

[46] Rodriguez-Perez MA, Gopal H, Adeleke MA, De Luna-Santillana EJ, Gurrola-Reyes JN, Guo X (2013). Detection of *Onchocerca volvulus* in Latin American black flies for pool screening PCR using high-throughput automated DNA isolation for transmission surveillance. Parasitol Res.

[47] Gopal H, Hassan HK, Rodriguez-Perez MA, Toe LD, Lustigman S, Unnasch TR (2012). Oligonucleotide based magnetic bead capture of *Onchocerca volvulus* DNA for PCR pool screening of vector black flies. PLoS Negl Trop Dis.

[48] Guevara AG, Vieira JC, Lilley BG, Lopez A, Vieira N, Rumbea J (2003). Entomological evaluation by pool screen polymerase chain reaction of *Onchocerca volvulus* transmission in Ecuador following mass Mectizan distribution. Am J Trop Med.

[49] Rodriguez-Perez MA, Katholi CR, Hassan HK, Unnasch TR (2006). Large-scale entomologic assessment of *Onchocerca volvulus* transmission by poolscreen PCR in Mexico. Am J Trop Med.

[50] Zhang X, Lowe SB, Gooding JJ (2014). Brief review of monitoring methods for loop-mediated isothermal amplification (LAMP). Biosens Bioelectron.

[51] Seyrig G, Stedtfeld RD, Tourlousse DM, Ahmad F, Towery K, Cupples AM (2015). Selection of fluorescent DNA dyes for real-time LAMP with portable and simple optics. J Microbiol Methods.

[52] Nagai S, Yamamoto K, Hata N, Itakura S (2012). Study of DNA extraction methods for use in loop-mediated isothermal amplification detection of single resting cysts in the toxic dinoflagellates *Alexandrium tamarense* and *A. catenella*. Mar Genomics.

[53] Sun Y, Zhao L, Zhao M, Zhu R, Deng J, Wang F (2014). Four DNA extraction methods used in loop-mediated isothermal amplification for rapid adenovirus detection. J Virol Methods.

[54] Creecy A, Russ PK, Solinas F, Wright DW, Haselton FR (2015). Tuberculosis biomarker extraction and isothermal amplification in an integrated diagnostic device. PLoS One.

