# Supplementary figures and images for: An isothermal DNA amplification method for detection of Onchocerca volvulus infection in skin biopsies
Source: Parasit Vectors. 2016 Dec 1;9:624. doi: 10.1186/s13071-016-1913-7 (PMC5134125; doi:10.1186/s13071-016-1913-7)

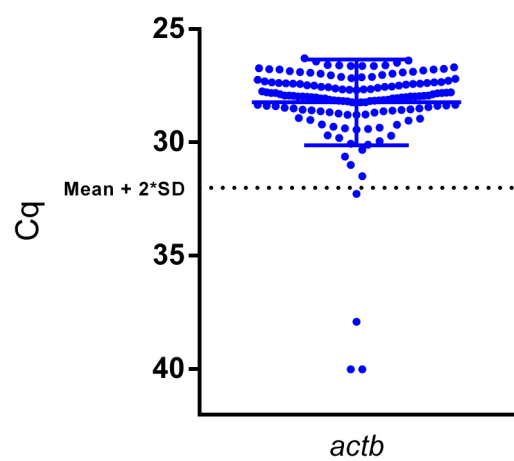

Supplement: Additional file 1: Figure S1. — Cq values of actb qPCR assay on the 150 gDNA samples. Mean and standard deviation are indicated, as well as the line indicating mean + 2 standard deviations. The four samples that had Cq values above this cut-off were excluded in further analyses. (PDF 29 kb) [file 13071_2016_1913_MOESM1_ESM.pdf]

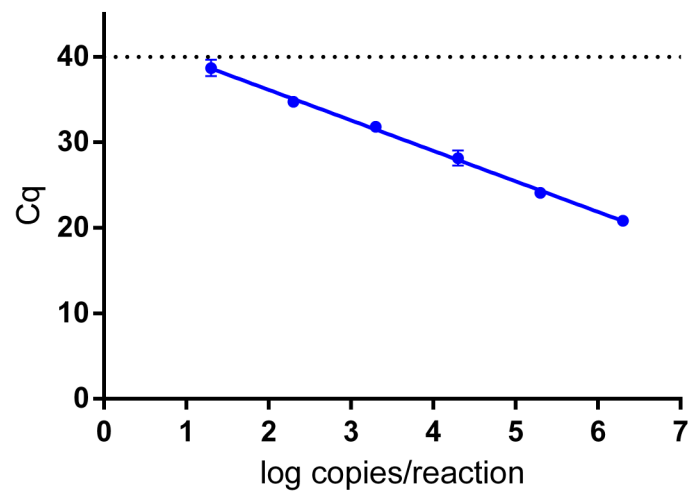

Supplement: Additional file 2: Figure S2. — Calibration curve of the O-150 qPCR assay. Calibration samples have been analyzed in 4-fold. Geometric mean and 95% confidence interval are indicated. (PDF 34 kb) [file 13071_2016_1913_MOESM2_ESM.pdf]
